# Benzoxazole derivatives: design, synthesis and biological evaluation

**DOI:** 10.1186/s13065-018-0459-5

**Published:** 2018-08-12

**Authors:** Saloni Kakkar, Sumit Tahlan, Siong Meng Lim, Kalavathy Ramasamy, Vasudevan Mani, Syed Adnan Ali Shah, Balasubramanian Narasimhan

**Affiliations:** 1 Faculty of Pharmaceutical Sciences, Maharshi Dayanand University, Rohtak, 124001 India; 2 Faculty of Pharmacy, Universiti Teknologi MARA (UiTM), Puncak Alam Campus, 42300 Bandar Puncak Alam, Selangor Darul Ehsan Malaysia; 3 Collaborative Drug Discovery Research (CDDR) Group, Pharmaceutical Life Sciences Community of Research, Universiti Teknologi MARA (UiTM), 40450 Shah Alam, Selangor Darul Ehsan Malaysia; 4 Department of Pharmacology and Toxicology, College of Pharmacy, Qassim University, Buraidah, 51452 Kingdom of Saudi Arabia; 5 Atta-ur-Rahman Institute for Natural Products Discovery (AuRIns), Universiti Teknologi MARA (UiTM), Puncak Alam Campus, 42300 Bandar Puncak Alam, Selangor Darul Ehsan Malaysia

**Keywords:** Benzoxazole, Synthesis, Antimicrobial, Anticancer, Characterization

## Abstract

**Background:**

A new series of benzoxazole analogues was synthesized and checked for their in vitro antibacterial, antifungal and anticancer activities.

**Results and discussion:**

The synthesized benzoxazole compounds were confirmed by IR, ^1^H/^13^C-NMR, mass and screened for their in vitro antimicrobial activity against Gram-positive bacterium: *Bacillus subtilis*, four Gram-negative bacteria: *Escherichia coli*, *Pseudomonas aeruginosa*, *Klebsiella pneumoniae*, *Salmonella typhi* and two fungal strains: *Candida albicans* and *Aspergillus niger* using tube dilution technique and minimum inhibitory concentration (MIC) was noted in µM and compared to ofloxacin and fluconazole. Human colorectal carcinoma (HCT116) cancer cell line was used for the determination of in vitro anticancer activity (IC_50_ value) by Sulforhodamine B assay using 5-fluorouracil as standard drug.

**Conclusion:**

The performed study indicated that the compounds **1**, **10**, **13**, **16**, **19**, **20** and **24** had highest antimicrobial activity with MIC values comparable to ofloxacin and fluconazole and compounds **4**, **6**, **25** and **26** had best anticancer activity in comparison to 5-fluorouracil.
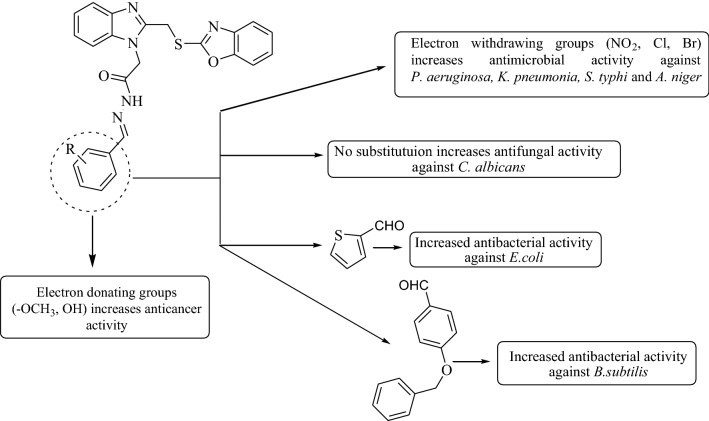

## Background

**Fig. 1:**
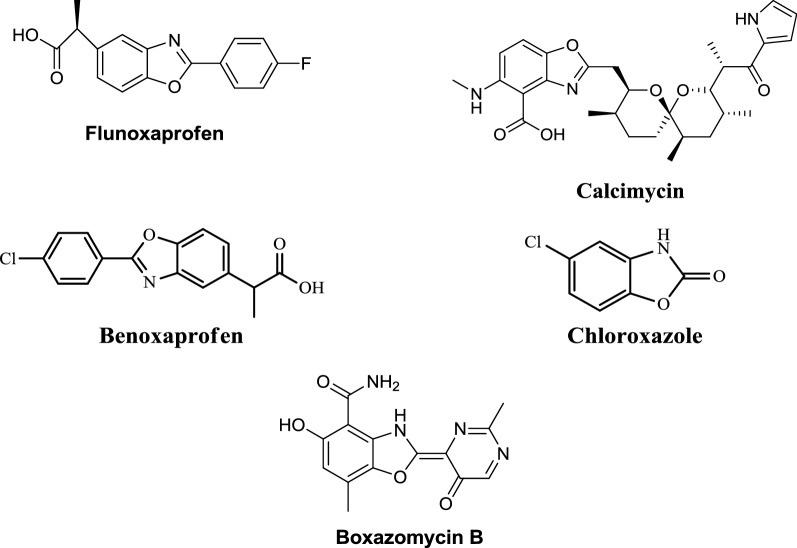
Marketed drugs containing benzoxazole

**Fig. 2:**
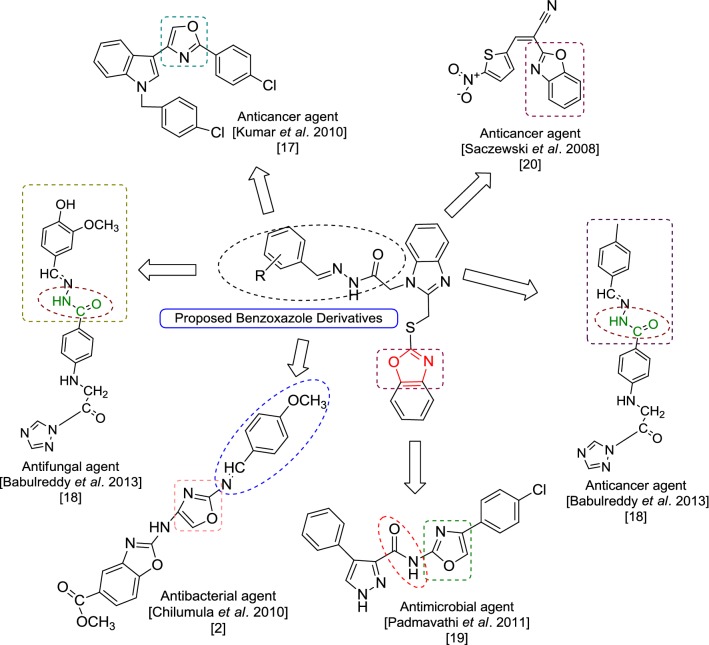
Design of benzoxazole molecules for antimicrobial and anticancer potential based on literature

A great number of deaths are occurring throughout the world because of infectious diseases [1]. It has been observed that there is a rapid increase in multi drug resistant infections these days which are causing a rise in various public health problems. There are number of diseases which are now hard to treat with traditional antibiotics drugs and clinicians have to depend on limited drugs such as vancomycin [2]. Because of this there is an increased demand to develop newer antimicrobial agents [3]. One of the most dangerous diseases in the world is cancer and irrespective of so much medical advancement, cancer remains the second leading cause of death in developing as well as developed countries. Although chemotherapy is mostly used for treating cancer, the failure of available chemotherapeutics to treat cancer underscores the need of developing new chemical entities [4]. Human colorectal cancer (CRC) has poor prognosis and is the third most commonly diagnosed malignancies. Therapy is very much required with better efficacy, less adverse effects and improved survival rates [5]. Benzoxazole derivatives have gained a lot of importance in the past few years because of their use in intermediates for the preparation of new biological materials. Benzoxazoles are prominent in medicinal chemistry due to their wide spectrum of pharmacological activities such as antibacterial [2], antifungal [6], anticancer [7], anti-inflammatory [8], antimycobacterial [9], antihistamine [10], antiparkinson [11], inhibition of hepatitis C virus [12], 5-HT_3_ antagonistic effect [13], melatonin receptor antagonism [14], amyloidogenesis inhibition [15] and Rho-kinase inhibition [16]. A number of marketed drugs (Fig. 1) are available having benzoxazole as core active moiety like, *nonsteroidal anti*-*inflammatory drug* (*NSAID*)—flunoxaprofen, benoxaprofen, *antibiotic*—calcimycin, *antibacterial*—boxazomycin B, *muscle relaxant*—chloroxazone. Prompted by the above findings (Fig. 2) in the present study, we hereby report the synthesis, antimicrobial and anticancer activities of a series of benzoxazole derivatives.

## Results and discussion

### Chemistry

**Scheme 1:**
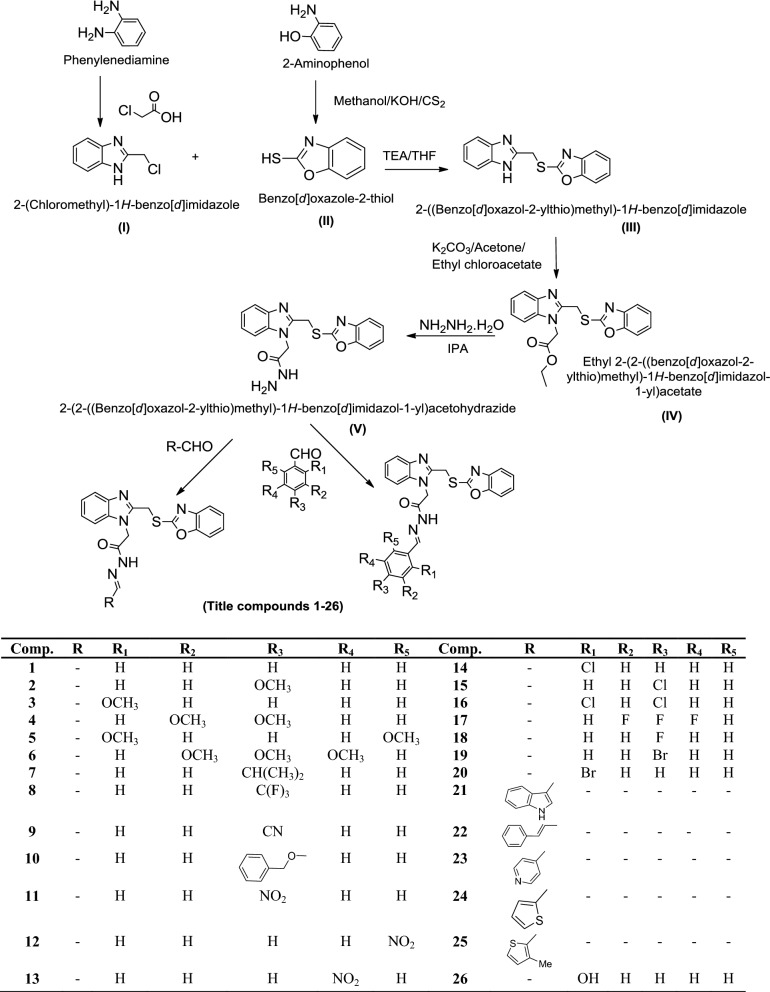
Synthesis of 2-(2-((benzo[*d*]oxazol-2-ylthio)methyl)-1*H*-benzo[*d*]imidazol-1-yl)acetohydrazide derivatives

The method to synthesize the designed benzoxazole derivatives is given in Scheme 1. Initially, 2-(chloromethyl)-1*H*-benzo[*d*]imidazole (**I**) was synthesized by the reaction of *ortho* phenylenediamine, chloroacetic acid and hydrochloric acid. Benzo[*d*]oxazole-2-thiol (**II**) was synthesized by the reaction of methanolic solution of 2-aminophenol with potassium hydroxide, followed by the addition of carbon-di-sulfide. A mixture of **I** and **II** was stirred in the presence of triethylamine so as to obtain 2-(((1*H*-benzimidazol-2-yl) methyl)thio)benzoxazole (**III**). To a mixture of **III** and anhydrous potassium carbonate in dry acetone, ethyl chloroacetate was added so as to get ethyl 2-(2-((benzoxazol-2-ylthio)methyl)-1*H*-benzimidazol-1-yl)acetate **(IV**). Further reaction of **IV** with hydrazine hydrate yielded 2-(2-((benzoxazol-2-ylthio) methyl)-1*H*-benzimidazol-1-yl) acetohydrazide (**V**). Finally reaction of **V** with various substituted aldehydes gave the title compounds (**1**–**26**). The physicochemical properties of newly synthesized compounds are given in Table 1. The molecular structures of the synthesized compounds (**1**–**26**) were determined by IR (ATR, cm^−1^), ^1^H/^13^C-NMR (DMSO-*d*_6,_ 400 MHz, ppm) and mass spectral studies.

**Table 1:** The physicochemical properties of synthesized benzoxazole derivatives (**1**–**26**)

Comp.	Molecular formula	Molecular structure	M. Wt.	M. Pt.	R_*f*_ value^a^	% yield
**1**.	C_24_H_19_N_5_O_2_S		441.50	180–182	0.58	95
**2**.	C_25_H_21_N_5_O_3_S		471.53	242–244	0.52	93
**3**.	C_25_H_21_N_5_O_3_S		471.53	224–225	0.51	86
**4**.	C_26_H_23_N_5_O_4_S		501.56	233–235	0.55	85
**5**.	C_26_H_23_N_5_O_4_S		501.56	256–258	0.55	82
**6**.	C_27_H_25_N_5_O_5_S		531.58	263–265	0.56	85
**7**.	C_27_H_25_N_5_O_2_S		483.58	184–186	0.60	87
**8**.	C_25_H_18_F_3_N_5_O_2_S		509.50	190–192	0.53	88
**9**.	C_25_H_18_N_6_O_2_S		466.51	278–280	0.51	91
**10**.	C_31_H_25_N_5_O_3_S		547.63	261–263	0.55	85
**11**.	C_24_H_18_N_6_O_4_S		486.50	283–285	0.48	92
**12**.	C_24_H_18_N_6_O_4_S		486.50	243–245	0.47	91
**13**.	C_24_H_18_N_6_O_4_S		486.50	259–261	0.45	95
**14**.	C_24_H_18_ClN_5_O_2_S		475.95	200–202	0.49	92
**15**.	C_24_H_18_ClN_5_O_2_S		475.95	237–239	0.44	92
**16**.	C_24_H_17_Cl_2_N_5_O_2_S		510.39	257–259	0.46	94
**17**.	C_24_H_16_F_3_N_5_O_2_S		495.47	178–180	0.52	83
**18**.	C_24_H_18_FN_5_O_2_S		459.49	184–186	0.53	93
**19**.	C_24_H_18_BrN_5_O_2_S		520.40	247–249	0.51	89
**20**.	C_24_H_18_BrN_5_O_2_S		520.40	207–209	0.52	83
**21**.	C_26_H_20_N_6_O_2_S		480.54	283–285	0.45	91
**22**.	C_26_H_21_N_5_O_2_S		467.54	187–189	0.48	92
**23**.	C_23_H_18_N_6_O_2_S		442.49	186–188	0.42	90
**24**.	C_22_H_17_N_5_O_2_S_2_		447.53	205–207	0.49	91
**25**.	C_23_H_19_N_5_O_2_S_2_		461.55	218–220	0.52	93
**26**.	C_24_H_19_N_5_O_3_S		457.50	192–194	0.45	95

^a^TLC mobile phase: chloroform: methanol (9:1)

The presence of IR absorption band at 3214 cm^−1^ in the spectral data of synthesized derivatives (**26**) corresponds to the group Ar–OH. The C–Br stretching of aromatic bromo compounds shows band around 705 cm^−1^ (**19** and **20**). The presence of Ar–NO_2_ group in compounds (**11**, **12** and **13**) was indicated by the appearance of asymmetric Ar–NO_2_ stretches in the scale of 1347–1339 cm^−1^. Arylalkyl ether category (Ar-OCH_3_) present in the compounds **2**, **3**, **4**, **5** and **6** shows IR absorption stretching at 3053–2835 cm^−1^. In case of halogen group Ar–Cl vibration appears at 747–740 cm^−1^ whereas existence of Ar–F group in compounds **8**, **17** and **18** was indicated by appearance of Ar–F stretches at 1383–1119 cm^−1^. The presence of IR stretching at 759–660 cm^−1^ reflected the presence of C–S group. The presence of CO–NH group is reflected by the presence of absorption bands at 1629–1605 cm^−1^ whereas the absorption bands at 3213–2919 cm^−1^, 1496–1452 cm^−1^ and 1688–1654 cm^−1^ corresponds to the presence of C–H, C=C and C=N group respectively. In case of ^1^H-NMR spectra the presence of multiplet signals between 6.85 and 8.83 ppm reflected the presence of aromatic protons in synthesized derivatives. The compound **26** showed singlet at 4.6 ppm because of the presence of OH of Ar–OH. The appearance of singlet at 7.01–8.24 ppm, 7.49–8.26 ppm, 4.61–4.63 ppm and 4.57–4.59 ppm is due to the existence of –CONH, N=CH, N–CH_2_ and CH_2_–S groups respectively. Compound **7** showed doublet around 1.22 ppm due to existence of isopropyl group at *para* position. Compounds **2**, **3**, **4**, **5** and **6** showed singlet at range of 3.72–3.81 ppm due to presence of OCH_3_ of Ar–OCH_3_. Finally, DMSO-*d*6 was used for recording the ^13^C-NMR spectra of benzoxazole derivatives and it was observed that the spectral signals and proposed molecular structure of the prepared compounds showed good agreement.

#### Antimicrobial activity

**Fig. 3:**
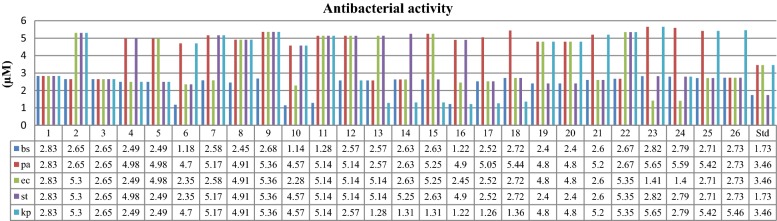
Antibacterial screening results against Gram positive and Gram negative species

**Fig. 4:**
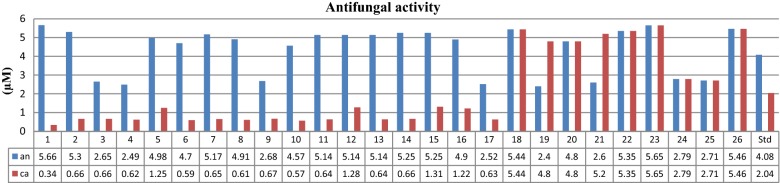
Antifungal screening results against fungal species

The screening of antibacterial and antifungal activity of the synthesized derivatives was done by tube dilution method [21] and the results are shown in Table 2 as well as Figs. 3 and 4. The study revealed that the prepared derivatives showed moderate to good antimicrobial activity against various microbial strains used. Particularly, compounds **1**, **10**, **13**, **16**, **19**, **20** and **24** have shown better antimicrobial activity than the standards ofloxacin and fluconazole. Compound **10** (MIC_*bs*_ = 1.14 × 10^−3^ µM) was found to be most effective against *B. subtilis.* Compound **24** (MIC_*ec*_ = 1.40 × 10^−3^ µM) was found to be active against *E. coli*, compound **13** (MIC_*pa*_ = 2.57 × 10^−3^ µM) against *P. aeruginosa*, compounds **19** and **20** (MIC_*st*_ = 2.40 × 10^−3^ µM) against *S. typhi*, compound **16** (MIC_*kp*_ = 1.22 × 10^−3^ µM) against *K. pneumonia*. The results of antifungal activity indicated that compound **19** (MIC_*an*_ = 2.40 × 10^−3^ µM) was most potent against *A. niger* and compound **1** (MIC_*ca*_ = 0.34 × 10^−3^ µM) was most effective against *C. albicans.* The other derivatives showed average to poor antimicrobial activity against all seven species.

**Table 2:** In vitro antimicrobial and anticancer screening of the synthesized derivatives (**1**–**26**)

Comp. no.	Antimicrobial screening (MIC = ×10^−3^ µM)							Anticancer screening (IC_50_ = µM)
	BS	PA	EC	ST	KP	AN	CA	HCT-116
1	2.83	2.83	2.83	2.83	2.83	5.66	0.34	192.5
2	2.65	2.65	5.30	5.30	5.30	5.30	0.66	84.8
3	2.65	2.65	2.65	2.65	2.65	2.65	0.66	> 212.1
4	2.49	4.98	2.49	4.98	2.49	2.49	0.62	39.9
5	2.49	4.98	4.98	2.49	2.49	4.98	1.25	> 199.4
6	1.18	4.70	2.35	2.35	4.70	4.70	0.59	24.5
7	2.58	5.17	2.58	5.17	5.17	5.17	0.65	> 206.8
8	2.45	4.91	4.91	4.91	4.91	4.91	0.61	> 196.3
9	2.68	5.36	5.36	5.36	5.36	2.68	0.67	> 214.4
10	1.14	4.57	2.28	4.57	4.57	4.57	0.57	> 182.6
11	1.28	5.14	5.14	5.14	5.14	5.14	0.64	> 205.5
12	2.57	5.14	5.14	5.14	2.57	5.14	1.28	> 205.5
13	2.57	2.57	5.14	5.14	1.28	5.14	0.64	> 205.5
14	2.63	2.63	2.63	5.25	1.31	5.25	0.66	> 210.1
15	2.63	5.25	5.25	2.63	1.31	5.25	1.31	> 210.1
16	1.22	4.90	2.45	4.90	1.22	4.90	1.22	> 195.9
17	2.52	5.05	2.52	2.52	1.26	2.52	0.63	> 201.8
18	2.72	5.44	2.72	2.72	1.36	5.44	5.44	78.3
19	2.40	4.80	4.80	2.40	4.80	2.40	4.80	> 192.2
20	2.40	4.80	4.80	2.40	4.80	4.80	4.80	> 192.2
21	2.60	5.20	2.60	2.60	5.20	2.60	5.20	> 208.1
22	2.67	2.67	5.35	5.35	5.35	5.35	5.35	70.6
23	2.82	5.65	1.41	2.82	5.65	5.65	5.65	> 226
24	2.79	5.59	1.40	2.79	2.79	2.79	2.79	96.1
25	2.71	5.42	2.71	2.71	5.42	2.71	2.71	45.5
26	2.73	2.73	2.73	2.73	5.46	5.46	5.46	35.6
Ofloxacin	1.73	3.46	3.46	1.73	3.46	–	–	–
Fluconazole	–	–	–	–	–	4.08	2.04	–
5-FU	–	–	–	–	–	–	–	29.2

#### Anticancer activity

Human colorectal carcinoma [HCT-116 (ATCC CCL-247)] cancer cell line was used for evaluating the anticancer activity of the prepared benzoxazole compounds using Sulforhodamine B (SRB) assay [22]. 5-Fluorouracil was used as standard drug and the results are shown in Table 2. The results indicated that the compound **6** (IC_50_ = 24.5 µM) exhibited the best anticancer activity in comparison with the standard drug (IC_50_ = 29.2 µM) whereas the compounds **4** and **26** displayed IC_50_ values closer to the reference drug (39.9 µM and 35.6 µM, respectively).

#### SAR (structure activity relationship) studies

**Fig. 5:**
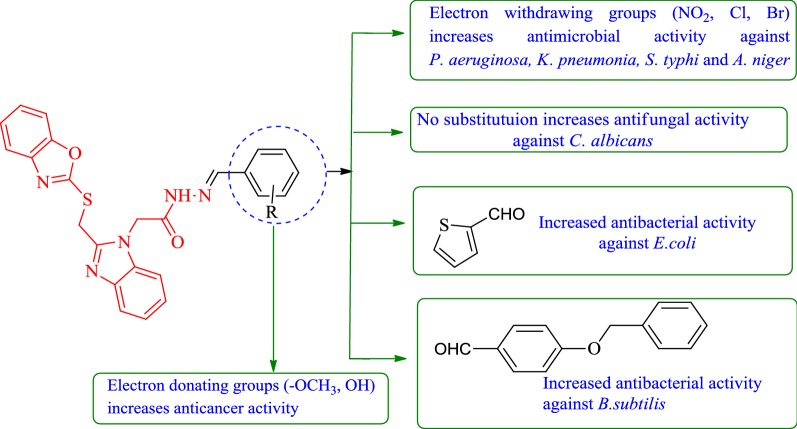
Structure activity relationship of the synthesized compounds

The structure–activity relationship of the synthesized benzoxazole derivatives with their antibacterial and anticancer activity results is summarized in Fig. 5.

- The substitution of aromatic aldehydes with di-methoxy (compound **4**) and tri-methoxy groups (compound **6**) improved the anticancer activity of prepared derivatives.
- Presence of *ortho* hydroxy group (compound **26**) improved the anticancer activity.
- Presence of unsubstituted benzylidene hydrazide (compound **1**) in synthesized oxazole derivatives improved the antifungal activity against *C. albicans*.
- Using (methoxymethyl)benzene (compound **10**) enhanced the antibacterial activity against *B. subtilis*.
- Presence of electron withdrawing groups (compounds **13**, **16**, **19** and **20**) improved the antimicrobial activity against *P. aeruginosa, K. pneumonia, S. typhi* and *A. niger.*
- Substitution of five member cyclic aldehyde i.e., thiophene (compound **24**) improved the antibacterial activity of benzoxazole derivatives against *E. coli*.

### Experimental part

The analytical grade chemicals procured from commercial sources were used as such without further purification. Thin-layer chromatography on 0.25 mm silica gel (Merck) plates was performed for monitoring the progress of reaction, using chloroform and methanol as mobile phase in ratio of 9:1 and exposure to iodine vapours helped in observing the spots. Open capillary tube was used for determining the melting points of synthesized compounds. Bruker 12060280, software: OPUS 7.2.139.1294 spectrometer was used for recording infrared spectrum (ATR). Bruker Avance III 600 NMR spectrometer was used for recording ^1^H and ^13^C NMR spectra in appropriate deuterated solvents and are expressed in parts per million (ppm) downfield from tetramethylsilane (internal standard). NMR data are given as multiplicity (s, singlet; d, doublet; t, triplet; m, multiplet) and number of protons. Perkin-Elmer 2400 C, H and N analyzer was utilized for the elemental analysis of the new synthesized compounds. All the compounds gave C, H and N analysis within ± 0.4% of the theoretical results. Mass spectra were obtained on Waters Micromass Q-ToF Micro instrument. The physicochemical and spectral data of the prepared compounds helped in their characterization.

#### Procedure for synthesis of benzoxazole derivatives (2-(2-((benzoxazol-2-ylthio) methyl)-1H-benzimidazol-1-yl) acetohydrazide)

##### Step 1: Synthesis of 2-(chloromethyl)-1H-benzo[d]imidazole (I)

Phenylenediamine (5.4 g), chloroacetic acid (7.1 g) and 4 N hydrochloric acid were refluxed for 16 h, the mixture was then allowed to stand overnight, filtered and diluted with 100 ml of water, cooled and carefully neutralized with solid sodium bicarbonate. The yellow solid was filtered, washed well with water, recrystallized with ethanol and dried to give the title compound (Yield: 80%). MP: 157–159 °C.

##### Step 2: Synthesis of benzo[d]oxazole-2-thiol (II)

A mixture of 2-aminophenol (1.1 g) in methanol (15 ml) was prepared to which potassium hydroxide (0.7 g) in water (3 ml) was added, followed by the addition of carbon-di-sulfide (0.9 ml). Resulting solution was refluxed at 65 °C for 5 h. After the completion of reaction, the mixture was poured in water, which was neutralized with concentrated hydrochloric acid. Solid separated was filtered and washed with hexane, recrystallized with ethanol and dried to afford the pure compound (Yield: 90%). MP: 168–170 °C.

##### Step 3: Synthesis of 2-(((1H-benzimidazol-2-yl) methyl)thio)benzoxazole (III)

A mixture of 2-(chloromethyl)-1*H*-benzimidazole (**1)** (1.66 g) and benzoxazole-2-thiol (**II**) (1.51 g) in dry THF (30 ml) was stirred in the presence of triethylamine (2 ml) for 6 h at room temperature. The reaction was monitored by TLC (chloroform: methanol/9:1, R_*f*_: 0.82). After the completion of reaction, THF was removed and ice cold water (30 ml) was added to the residue with stirring. The solid precipitated was filtered, washed with water followed by hexane, recrystallized with ethanol and dried to afford crude product **III** (2.5 g, 88%). MP: 181–183 °C.

##### Step 4: Synthesis of ethyl 2-(2-((benzoxazol-2-ylthio)methyl)-1H-benzimidazol-1-yl)acetate (IV)

A mixture of 2-(((1*H*-benzimidazol-2-yl)methyl)thio)benzoxazole (**III)** (2.8 g) and anhydrous potassium carbonate (1 g) in dry acetone (15 ml) was prepared to which ethyl chloroacetate (1.2 ml) was added and the mixture was stirred for 8 h at room temperature. The reaction was monitored by TLC (TLC System: chloroform: methanol/9:1, R_*f*_: 0.65). The resulting solution was then evaporated and solid obtained was suspended in cold water with stirring, which was then filtered, washed with water, recrystallized with ethanol and dried to give desired product **IV** (Yield: 3.1 g, 85%). MP: 163–165 °C.

##### Step 5: Synthesis of 2-(2-((benzoxazol-2-ylthio) methyl)-1H-benzimidazol-1-yl) acetohydrazide (V)

A suspension of ethyl 2-(2-((benzoxazol-2-ylthio)methyl)-1*H*-benzimidazol-1-yl)acetate (**IV**) (3.57 g) in isopropyl alcohol (30 ml) was added with hydrazine hydrate (98%, 5 ml) and was stirred at room temperature for 1 h. After the completion of reaction as indicated by TLC (chloroform: methanol/9:1, R_*f*_: 0.4), the reaction mixture was poured into ice cold water and the precipitated solid was filtered, washed with cold isopropyl alcohol and recrystallized with ethanol to give compound **V** as white solid (2.9 g, 82%). MP: 236–238 °C.

##### Step 6: Synthesis of final derivatives (1–26)

A solution of 2-(2-((benzoxazol-2-ylthio)methyl)-1*H*-benzimidazol-1-yl) acetohydrazide (**V**) (0.71 g) in acetic acid (5 ml) was added with corresponding substituted aldehydes The reaction mixture was stirred at room temperature for 30 min. After completion of the reaction as monitored by TLC (chloroform: methanol/9:1), the solution was poured in ice cold water and stirred for 30 min at room temperature. Solid separated out was then filtered, washed with water followed by isopropyl alcohol and recrystallized with ethanol to give pure product.

#### Spectral data of intermediates and final compounds (1–26)

##### Intermediate I

IR: 3048 (C–H str., aromatic), 1456 (C=C str., aromatic), 1662 (C=N, N=CH str.), 1189 (C–H str., –CH_2_), 745 (C–Cl str., Cl); ^1^H-NMR: 7.35–7.76 (m, 4H, ArH), 4.67 (s, 1H, –NH of imidazole), 4.52 (s, 2H, –CH_2_); ^13^C-NMR: 140.8, 137.9, 122.5, 114.6, 40.7; MS ES + (ToF): *m/z* 167 [M^+^+1]; CHN: Calc. C_8_H_7_ClN_2_: C, 57.67; H, 4.23; N, 16.81; Found: C, 57.72; H, 4.35; N, 16.97.

##### Intermediate II

IR: 3072 (C–H str., aromatic), 1462 (C=C str., aromatic), 1658 (C=N, N=CH str.), 1183 (C–O–C str. of oxazole), 2498 (–SH str.); ^1^H-NMR: 7.32 (m, 4H, ArH), 3.61 (s, 1H, –SH); ^13^C-NMR: 178.3, 151.2, 142.7, 124.4, 118.2, 111.7; MS ES + (ToF): *m/z* 152 [M^+^+1]; CHN: Calc. C_7_H_5_NOS: C, 64.04; H, 3.94; N, 14.94; Found: C, 64.09; H, 3.98; N, 14.97.

##### Intermediate III

IR: 3046 (C–H str., aromatic), 1485 (C=C str., aromatic), 1670 (C=N, N=CH str.), 1243 (C–N str.), 687 (CH_2_S, C–S str.), 1189 (C–O–C str. of oxazole); ^1^H-NMR: 7.31–7.70 (m, 8H, ArH), 3.61 (s, 2H, –CH_2_S), 4.88 (s, 1H, –NH of imidazole); ^13^C-NMR: 163.3, 151.3, 141.1, 124.6, 124.4, 118.3, 110.2, 38.8; MS ES + (ToF): *m/z* 282 [M^+^+1]; CHN: Calc. C_15_H_11_N_3_OS: C, 64.04; H, 3.94; N, 14.94; Found: C, 64.09; H, 3.98; N, 14.97.

##### Intermediate IV

IR: 3078 (C–H str., aromatic), 1475 (C=C str., aromatic), 1668 (C=N, N=CH str.), 1249 (C–N str.), 689 (CH_2_S, C–S str.), 1197 (C–O–C str. of oxazole), 3945 (C–H str., –CH_3_), 1782 (C=O str.), 2745 (C–H str., –OC_2_H_5_); ^1^H-NMR: 7.46–7.72 (m, 8H, ArH), 4.59 (s, 2H, –CH_2_S), 4.62 (s, 2H, –NCH_2_), 3.97 (s, 2H, –CH_2_), 1.92 (s, 3H, –CH_3_); ^13^C-NMR:164.7, 151.1, 141.8, 139.8, 132.9, 124.9, 124.4, 119.3, 114.4, 110.9, 55.2, 29.5; MS ES + (ToF): *m/z* 368 [M^+^+1]; CHN: Calc. C_19_H_17_N_3_O_3_S: C, 62.11; H, 4.66; N, 11.44; Found: C, 62.16; H, 4.72; N, 11.49.

##### Intermediate V

IR: 3031 (C–H str., aromatic), 1472 (C=C str., aromatic), 1674 (C=N, N=CH str.), 1240 (C–N str.), 694 (CH_2_S, C–S str.), 1194 (C–O–C str. of oxazole), 1624 (CONH str., amide), 1778 (C=O str.), 3392 (C–NH_2_ str.); ^1^H-NMR: 7.41–7.78 (m, 8H, ArH), 4.57 (s, 2H, –NCH_2_), 7.89 (s, 1H, –NH), 4.24 (s, 2H, –CH_2_S), 2.51 (s, 2H, –NH_2_); ^13^C-NMR: 167.9, 151.1, 141.7, 139.8, 132.8, 124.8, 124.4, 119.3, 113.7, 110.9, 32.3, 29.7; MS ES + (ToF): *m/z* 354 [M^+^+1]; CHN: Calc. C_17_H_15_N_5_O_2_S: C, 57.78; H, 4.28; N, 19.82; Found: C, 57.84; H, 4.34; N, 19.92.

##### Compound 1

IR: 3062 (C–H str., aromatic), 1490 (C=C str., aromatic), 1669 (C=N, N=CH str.), 1245 (C–N str.), 697 (CH_2_S, C–S str.), 1196 (C–O–C str. of oxazole), 1621 (CONH str., amide); ^1^H-NMR: 7.34–7.69 (m, 13H, ArH), 8.15 (s, 1H, N=CH–Ar), 4.63 (s, 2H, –NCH_2_), 7.95 (s, 1H, –NH), 4.59 (s, 2H, –CH_2_S); ^13^C-NMR: 170.4, 165, 151.1, 143.1, 141.7, 139.8, 134.1, 133.9, 130.1,129.7,128.7, 124.9, 124.4, 119.7, 113.7, 110.9, 33.3, 29.5; MS ES + (ToF): *m/z* 442 [M^+^+1]; CHN: Calc. C_24_H_19_N_5_O_2_S: C, 65.29; H, 4.34; N, 15.86; Found: C, 65.49; H, 4.40; N, 15.92.

##### Compound 2

IR: 3211 (C–H str., aromatic), 1455 (C=C str., aromatic), 1666 (C=N, N=CH str.), 1252 (C–N str.), 705 (CH_2_S, C–S str.), 1196 (C–O–C str. of oxazole), 1624 (CONH str., amide), 3053 (C–H str., –OCH_3_); ^1^H-NMR: 6.88–7.79 (m, 12H, ArH), 8.25 (s, 1H, N=CH–Ar), 4.62 (s, 2H, –NCH_2_), 8.08 (s, 1H, –NH), 4.59 (s, 2H, –CH_2_S), 3.77 (s, 3H, –OCH_3_); ^13^C-NMR: 170.2, 164.7, 151.1, 143.1, 141.8, 139.8, 132.9, 131.7, 126.5, 124.9, 124.4, 119.3, 114.4, 114.2, 110.9, 55.2, 33.3, 29.5; MS ES + (ToF): *m/z* 472 [M^+^+1]; CHN: Calc. C_25_H_21_N_5_O_3_S: C, 63.68; H, 4.49; N, 14.85; Found: C, 63.75; H, 4.54; N, 14.92.

##### Compound 3

IR: 3053 (C–H str., aromatic), 1456 (C=C str., aromatic), 1671 (C=N, N=CH str.), 1248 (C–N str.), 675 (CH_2_S, C–S str.), 1167 (C–O–C str. of oxazole), 1625 (CONH str., amide), 2941 (C–H str., –OCH_3_); ^1^H-NMR: 6.85–7.69 (m, 12H, ArH), 8.24 (s, 1H, N=CH–Ar), 4.62 (s, 2H, –NCH_2_), 7.89 (s, 1H, –NH), 4.58 (s, 2H, –CH_2_S), 3.81 (s, 3H, –OCH_3_); ^13^C-NMR: 170.3, 164.8, 151.1, 142.1, 141.8, 138.7, 132.8, 131.5, 131.2, 124.9, 124.4, 119.3, 113.7, 110.9, 55.6, 33.3, 29.5; MS ES + (ToF): *m/z* 472 [M^+^+1]; CHN: Calc. C_25_H_21_N_5_O_3_S: C, 63.68; H, 4.49; N, 14.85; Found: C, 63.78; H, 4.52; N, 14.88.

##### Compound 4

IR: 3011 (C–H str., aromatic), 1456 (C=C str., aromatic), 1661 (C=N, N=CH str.), 1244 (C–N str.), 703 (C–S str., CH_2_S), 1176 (C–O–C str. of oxazole), 1629 (CONH str., amide), 2880 (C–H str., –OCH_3_); ^1^H-NMR: 6.90–7.70 (m, 11H, ArH), 4.60 (s, 2H, –CH_2_S), 4.62 (s, 2H, –NCH_2_), 8.24 (s, 1H, N=CH–Ar), 8.05 (s, 1H, –NH), 3.77 (s, 6H, (–OCH_3_)_2_); ^13^C-NMR: 170.3, 164.7, 152.2, 150.3, 148.9, 143.2, 141.7, 139.8, 132.9, 126.7, 124.8, 124.4, 121.7, 119.3, 113.7, 110.9, 55.4, 33.34, 29.6; MS ES + (ToF): *m/z* 502 [M^+^+1]; CHN: Calc. C_26_H_23_N_5_O_4_S: C, 62.26; H, 4.62; N, 13.96; Found: C, 62.31; H, 4.72; N, 13.99.

##### Compound 5

IR: 3072 (C–H str., aromatic), 1456 (C=C str., aromatic), 1665 (C=N, N=CH str.), 1247 (C–N str.), 683 (C–S str., CH_2_S), 1168 (C–O–C str. of oxazole), 1626 (CONH str., amide), 2835 (C–H str., –OCH_3_); ^1^H-NMR: 6.93–7.76 (m, 11H, ArH), 4.59 (s, 2H, –CH_2_S), 4.62 (s, 2H, –NCH_2_), 8.26 (s, 1H, N=CH–Ar), 8.24 (s, 1H, –NH), 3.74 (s, 6H, (–OCH_3_)_2_); ^13^C-NMR: 170.5, 164.8, 153.1, 151.1, 142.1, 141.7, 138.6, 132.8, 124.9, 124.4, 122.5, 119.2, 116.8, 110.9, 110.8, 108.9, 55.3, 33.3, 29.6; MS ES + (ToF): *m/z* 502 [M^+^+1]; CHN: Calc. C_26_H_23_N_5_O_4_S: C, 62.26; H, 4.62; N, 13.96; Found: C, 62.33; H, 4.68; N, 13.98.

##### Compound 6

IR: 3208 (C–H str., aromatic), 1454 (C=C str., aromatic), 1657 (C=N, N=CH str.), 1238 (C–N str.), 704 (C–S str., CH_2_S), 1158 (C–O–C str. of oxazole), 1625 (CONH str., amide), 3002 (C–H str., –OCH_3_); ^1^H-NMR: 6.9–7.74 (m, 10H, ArH), 3.82 (s, 2H, –CH_2_S), 4.61 (s, 2H, –NCH_2_), 8.24 (s, 1H, N=CH–Ar), 8.07 (s, 1H, –NH), 3.72 (s, 9H, (–OCH_3_)_3_); ^13^C-NMR: 170.6, 164.9, 151.1, 148.2, 141.7, 138.8, 132.8, 129.4, 124.9, 124.4, 119.2, 113.7, 110.8, 104.2, 55.8, 33.3, 29.7; MS ES + (ToF): *m/z* 532 [M^+^+1]; CHN: Calc. C_27_H_25_N_5_O_5_S: C, 61.00; H, 4.74; N, 13.17; Found: C, 61.05; H, 4.78; N, 13.24.

##### Compound 7

IR: 3055 (C–H str., aromatic), 1455 (C=C str., aromatic), 1669 (C=N, N=CH str.), 1245 (C–N str.), 706 (C–S str., CH_2_S), 1165 (C–O–C str. of oxazole), 1622 (CONH str., amide), 3002 (C–H str., –OCH_3_); ^1^H-NMR: 7.2–7.79 (m, 12H, ArH), 4.59 (s, 2H, –CH_2_S), 4.62 (s, 2H, –NCH_2_), 8.25 (s, 1H, N=CH–Ar), 8.11 (s, 1H, –NH), 3.37 (s, 1H, –CH), 1.22 (d, 6H, (–CH_3_)_2_); ^13^C-NMR: 170.3, 164.9, 150.6, 150.3, 143.1, 141.8, 139.8, 132.9, 131.6, 127.1, 126.6, 124.9, 124.4, 119.3, 113.7, 110.9, 38.8, 33.2, 29.6, 23.6; MS ES + (ToF): *m/z* 484 [M^+^+1]; CHN: Calc. C_27_H_25_N_5_O_2_S: C, 67.06; H, 5.21; N, 14.48; Found: C, 67.09; H, 5.28; N, 14.52.

##### Compound 8

IR: 3213 (C–H str., aromatic), 1456 (C=C str., aromatic), 1670 (C=N, N=CH str.), 1242 (C–N str.), 682 (C–S str., CH_2_S), 1160 (C–O–C str. of oxazole), 1622 (CONH str., amide), 1119 (C–F); ^1^H-NMR: 7.39–7.68 (m, 12H, ArH), 4.59 (s, 2H, –CH_2_S), 4.63 (s, 2H, –NCH_2_), 8.21 (s, 1H, N=CH–Ar), 8.01 (s, 1H, –NH); ^13^C-NMR: 170.7, 165.3, 151.1, 144.9, 141.4, 138, 137.9, 132.8, 129.5, 125.3, 124.8, 124.4, 122.6, 119.2, 113.7, 110.9, 33.3, 29.6; MS ES + (ToF): *m/z* 510 [M^+^+1]; CHN: Calc. C_25_H_18_F_3_N_5_O_2_S: C, 58.93; H, 3.56; N, 13.75; Found: C, 58.99; H, 3.62; N, 13.78.

##### Compound 9

IR: 3085 (C–H str., aromatic), 1460 (C=C str., aromatic), 1673 (C=N, N=CH str.), 1239 (C–N str.), 709 (C–S str., CH_2_S), 1186 (C–O–C str. of oxazole), 1620 (CONH str., amide), 2227 (C≡N str., cyanide); ^1^H-NMR: 7.40–7.8 (m, 12H, ArH), 4.58 (s, 2H, –CH_2_S), 4.63 (s, 2H, –NCH_2_), 8.19 (s, 1H, N=CH–Ar), 7.98 (s, 1H, –NH); ^13^C-NMR: 170.8, 165.3, 151.1, 144.7, 141.7, 138.5, 138.3, 132.8, 127.5, 124.9, 124.4, 119.2, 113.7, 110.9, 33.3, 29.5; MS ES + (ToF): *m/z* 467 [M^+^+1]; CHN: Calc. C_25_H_18_N_6_O_2_S: C, 64.36; H, 3.89; N, 18.01; Found: C, 64.38; H, 3.93; N, 18.07.

##### Compound 10

IR: 3053 (C–H str., aromatic), 1456 (C=C str., aromatic), 1671 (C=N, N=CH str.), 1248 (C–N str.), 675 (C–S str., CH_2_S), 1167 (C–O–C str. of oxazole), 1625 (CONH str., amide); ^1^H-NMR: 6.96–7.78 (m, 17H, ArH), 4.58 (s, 2H, –CH_2_S), 4.62 (s, 2H, –NCH_2_), 8.24 (s, 1H, N=CH–Ar), 8.08 (s, 1H, –NH), 5.15 (s, 2H, –OCH_2_–Ar); ^13^C-NMR: 170.2, 164.7, 151.1, 142.9, 141.8, 139.8, 132.9, 128.6, 127.7, 126.8, 124.8, 124.4, 119.3, 115.1, 114.9, 110.9, 69.2, 31.6, 29.5; MS ES + (ToF): *m/z* 548 [M^+^+1]; CHN: Calc. C_31_H_25_N_5_O_3_S: C, 67.99; H, 4.60; N, 12.79; Found: C, 68.03; H, 4.64; N, 12.84.

##### Compound 11

IR: 2955 (C–H str., aromatic), 1456 (C=C str., aromatic), 1673 (C=N, N=CH str.), 1242 (C–N str.), 692 (C–S str., CH_2_S), 1164 (C–O–C str. of oxazole), 1624 (CONH str., amide), 1339 (N=O, Nitro); ^1^H-NMR: 7.40–8.23 (m, 12H, ArH), 4.59 (s, 2H, –CH_2_S), 4.63 (s, 2H, –NCH_2_), 8.13 (s, 1H, N=CH–Ar), 8.02 (s, 1H, –NH); ^13^C-NMR: 170.8, 165.4, 151.2, 150.9, 144.2, 141.7, 139.7, 132.8, 124.9, 123.9, 123.8, 119.2, 113.7, 110.8, 33.3, 29.6; MS ES + (ToF): *m/z* 487 [M^+^+1]; CHN: Calc. C_24_H_18_N_6_O_4_S: C, 59.25; H, 3.73; N, 17.27; Found: C, 59.16; H, 3.78; N, 17.33.

##### Compound 12

IR: 3064 (C–H str., aromatic), 1456 (C=C str., aromatic), 1672 (C=N, N=CH str.), 1244 (C–N str.), 701 (C–S str., CH_2_S), 1166 (C–O–C str. of oxazole), 1625 (CONH str., amide), 1342 (N=O, Nitro); ^1^H-NMR: 7.41–8.23 (m, 12H, ArH), 4.58 (s, 2H, –CH_2_S), 4.63 (s, 2H, –NCH_2_), 8.09 (s, 1H, N=CH–Ar), 8.00 (s, 1H, –NH); ^13^C-NMR: 172.1, 165.3, 151.1, 148.2, 142.1, 141.7, 138.5, 133.6, 132.8, 130.3, 125.3, 124.9, 124.4, 119.2, 113.7, 110.8, 33.3, 29.5; MS ES + (ToF): *m/z* 487 [M^+^+1]; CHN: Calc. C_24_H_18_N_6_O_4_S: C, 59.25; H, 3.73; N, 17.27; Found: C, 59.29; H, 3.65; N, 17.34.

##### Compound 13

IR: 3075 (C–H str., aromatic), 1453 (C=C str., aromatic), 1666 (C=N, N=CH), 1241 (C–N str.), 677 (C–S str., CH_2_S), 1167 (C–O–C str. of oxazole), 1624 (CONH str., amide), 1347 (N=O, Nitro); ^1^H-NMR: 7.38–8.48 (m, 12H, ArH), 4.59 (s, 2H, –CH_2_S), 4.63 (s, 2H, –NCH_2_), 8.11 (s, 1H, N=CH–Ar), 8.03 (s, 1H, –NH); ^13^C-NMR: 170.7, 165.3, 151.1, 148.2, 144.3, 141.7, 139.8, 135.8, 133.1, 130.2, 124.9, 124.2, 123.9, 119.2, 113.7, 110.9, 33.3, 29.5; MS ES + (ToF): *m/z* 487 [M^+^+1]; CHN: Calc. C_24_H_18_N_6_O_4_S: C, 59.25; H, 3.73; N, 17.27; Found: C, 59.30; H, 3.75; N, 17.30.

##### Compound 14

IR: 3058 (C–H str., aromatic), 1456 (C=C str., aromatic), 1670 (C=N, N=CH str.), 1246 (C–N str.), 660 (C–S str., CH_2_S), 1167 (C–O–C str.. of oxazole), 1625 (CONH str., amide), 747 (C–Cl str., Ar–Cl); ^1^H-NMR: 7.25–7.77 (m, 12H, ArH), 4.59 (s, 2H, –CH_2_S), 4.63 (s, 2H, –NCH_2_), 8.23 (s, 1H, N=CH–Ar), 7.94 (s, 1H, –NH); ^13^C-NMR: 170.6, 165.1, 151.1, 142.5, 141.7, 139.1, 133, 132.8, 129.8, 127.3, 124.9, 124.4, 119.2, 113.7, 110.8, 33.4, 29.5 MS ES + (ToF): *m/z* 476 [M^+^+1]; CHN: Calc. C_24_H_18_ClN_5_O_2_S: C, 60.56; H, 3.81; N, 14.71; Found: C, 60.58; H, 3.85; N, 14.73.

##### Compound 15

IR: 3082 (C–H str., aromatic), 1460 (C=C str., aromatic), 1670 (C=N str., N=CH str.), 1223 (C–N str.), 708 (C–S str., CH_2_S), 1186 (C–O–C str. of oxazole), 1619 (CONH str., amide), 745 (C–Cl str., Ar–Cl); ^1^H-NMR: 7.38–7.70 (m, 12H, ArH), 4.58 (s, 2H, –CH_2_S), 4.62 (s, 2H, –NCH_2_), 8.13 (s, 1H, N=CH–Ar), 7.93 (s, 1H, –NH); ^13^C-NMR: 170.53, 165, 151, 141.8, 141.7, 139.8, 134.4, 132.9, 128.8, 124.8, 124.4, 119.3, 113.7, 110.9, 33.3, 29.5; MS ES + (ToF): *m/z* 476 [M^+^+1]; CHN: Calc. C_24_H_18_ClN_5_O_2_S: C, 60.56; H, 3.81; N, 14.71; Found: C, 60.50; H, 3.87; N, 14.65.

##### Compound 16

IR: 2946 (C–H str., aromatic), 1454 (C=C str., aromatic), 1667 (C=N, N=CH str.), 1242 (C–N str.), 672 (C–S str., CH_2_S), 1167 (C–O–C str. of oxazole), 1623 (CONH str., amide), 740 (C–Cl str., Ar–Cl); ^1^H-NMR: 7.28–7.69 (m, 11H, ArH), 4.57 (s, 2H, –CH_2_S), 4.62 (s, 2H, –NCH_2_), 8.22 (s, 1H, N=CH–Ar), 7.90 (s, 1H, –NH); ^13^C-NMR: 170.6, 165.2, 151.1, 141.7, 141.5, 139.8, 138.1, 135, 134.7, 132.8, 130.3, 130.2, 127.7, 124.9, 124.4, 119.2, 113.7, 110.9, 33.4, 29.5; MS ES + (ToF): *m/z* 511 [M^+^+1]; CHN: Calc. C_24_H_17_Cl_2_N_5_O_2_S: C, 56.48; H, 3.36; N, 13.72; Found: C, 56.52; H, 3.44; N, 13.75.

##### Compound 17

IR: 3060 (C–H str., aromatic), 1452 (C=C str., aromatic), 1663 (C=N, N=CH str.), 1242 (C–N str.), 738 (C–S str., CH_2_S), 1169 (C–O–C str. of oxazole), 1625 (CONH str., amide), 1383 (C–F str., Ar–F); ^1^H-NMR: 7.39–7.75 (m, 10H, ArH), 4.58 (s, 2H, –CH_2_S), 4.62 (s, 2H, –NCH_2_), 8.23 (s, 1H, N=CH–Ar), 8.11 (s, 1H, –NH); ^13^C-NMR: 170.9, 165.3, 151.3, 151.1, 143.4, 141.7, 138.1, 132.8, 124.9, 124.4, 119.2, 113.7, 111.2, 110.8, 33.3, 29.5; MS ES + (ToF): *m/z* 496 [M^+^+1]; CHN: Calc. C_24_H_16_F_3_N_5_O_2_S: C, 58.18; H, 3.25; N, 11.50; Found: C, 58.10; H, 3.27; N, 11.53.

##### Compound 18

IR: 3130 (C–H str., aromatic), 1496 (C=C str., aromatic), 1671 (C=N, N=CH str.), 1267 (C–N str.), 706 (C–S str., CH_2_S), 1152 (C–O–C str. of oxazole), 1605 (CONH str., amide), 1351 (C–F str., Ar–F); ^1^H-NMR: 7.16–7.71 (m, 12H, ArH), 4.58 (s, 2H, –CH_2_S), 4.62 (s, 2H, –NCH_2_), 8.24 (s, 1H, N=CH–Ar), 8.14 (s, 1H, –NH); ^13^C-NMR: 170.4, 165.1, 151.1, 145.6, 141.7, 139.8, 132.8, 130.7, 129.2, 124.8, 124.4, 119.3, 113.7, 33.3, 29.5; MS ES + (ToF): *m/z* 460 [M^+^+1]; CHN: Calc. C_24_H_18_FN_5_O_2_S: C, 62.73; H, 3.95; N, 15.24; Found: C, 62.76; H, 3.98; N, 15.16.

##### Compound 19

IR: 3055 (C–H str., aromatic), 1485 (C=C str., aromatic), 1688 (C=N, N=CH str.), 1245 (C–N str.), 705 (C–S str., CH_2_S), 1188 (C–O–C str. of oxazole), 1626 (CONH str., amide), 705 (C–Br str., Ar–Br); ^1^H-NMR: 7.40–7.77 (m, 12H, ArH), 4.58 (s, 2H, –CH_2_S), 4.62 (s, 2H, –NCH_2_), 8.24 (s, 1H, N=CH–Ar), 8.11 (s, 1H, –NH); ^13^C-NMR: 170.5, 165.1, 151.1, 141.9, 141.7, 139.8, 133.3, 132.8, 131.7, 131.6, 125.3, 124.9, 124.4, 123.2, 119.2, 113.7, 110.9, 33.3, 29.5; MS ES + (ToF): *m/z* 521 [M^+^+1]; CHN: Calc. C_24_H_18_BrN_5_O_2_S: C, 55.39; H, 3.49; N, 13.46; Found: C, 55.42; H, 3.51; N, 13.50.

##### Compound 20

IR: IR: 3057 (C–H str., aromatic), 1458 (C=C str., aromatic), 1669 (C=N, N=CH str.), 1287 (C–N str.), 707 (C–S str., CH_2_S), 1247 (C–O–C str. of oxazole), 1626 (CONH str., amide), 707 (C–Br str., Ar–Br); ^1^H-NMR: 7.29–7.69 (m, 12H, ArH), 4.58 (s, 2H, –CH_2_S), 4.62 (s, 2H, –NCH_2_), 8.24 (s, 1H, N=CH–Ar), 7.91 (s, 1H, –NH); ^13^C-NMR: 170.6, 165.1, 151.1, 144.9, 141.5, 139.8, 133.1, 131.7, 131.3, 127.8, 124.9, 123.4, 123.1, 119.2, 113.7, 110.9, 33.4, 29.5; MS ES + (ToF): *m/z* 521 [M^+^+1]; CHN: Calc. C_24_H_18_BrN_5_O_2_S: C, 55.39; H, 3.49; N, 13.46; Found: C, 55.43; H, 3.42; N, 13.52.

##### Compound 21

IR: 3051 (C–H str., aromatic), 1453 (C=C str., aromatic), 1654 (C=N, N=CH str.), 1246 (C–N str.), 686 (C–S str., CH_2_S), 1159 (C–O–C str. of oxazole), 1619 (CONH str., amide), 3160 (N–H str., indole); ^1^H-NMR: 7.03–7.76 (m, 12H, ArH), 4.58 (s, 2H, –CH_2_S), 4.63 (s, 2H, –NCH_2_), 7.51 (s, 1H, N=CH–Ar), 7.01 (s, 1H, –NH); ^13^C-NMR: 169.4, 164.1, 151.1, 143.9, 141.7, 139.8, 136.9, 132.9, 130.3, 125.3, 124.9, 123.9, 122.5, 122.5, 120.4, 119.7, 119.3, 113.7, 111.1, 110.9, 32.2, 29.6; MS ES + (ToF): *m/z* 481 [M^+^+1]; CHN: Calc. C_26_H_20_N_6_O_2_S: C, 64.98; H, 4.20; N, 17.49; Found: C, 65.07; H, 4.25; N, 17.52.

##### Compound 22

IR: 3054 (C–H str., aromatic), 1453 (C=C str., aromatic), 1665 (C=N, N=CH str.), 1245 (C–N str.), 746 (C–S str., CH_2_S), 1202 (C–O–C str. of oxazole), 1624 (CONH str., amide), 1570 (conjugation); ^1^H-NMR: 7.29–7.76 (m, 13H, ArH), 4.58 (s, 2H, –CH_2_S), 4.61 (s, 2H, –NCH_2_), 7.51 (s, 1H, N=CH–Ar), 7.01 (s, 1H, –NH); ^13^C-NMR: 170.1, 164.8, 151.1, 141.7, 139.1, 138.6, 135.7, 128.7, 128.7, 126.9, 124.9, 124.4, 119.3, 113.7, 110.9, 33.3, 29.5; MS ES + (ToF): *m/z* 468 [M^+^+1]; CHN: Calc. C_26_H_21_N_5_O_2_S: C, 66.79; H, 4.53; N, 14.98; Found: C, 66.71; H, 4.58; N, 15.05.

##### Compound 23

IR: 3079 (C–H str., aromatic), 1460 (C=C str., aromatic), 1687 (C=N, N=CH str.), 1241 (C–N str.), 707 (C–S str., CH_2_S), 1186 (C–O–C str. of oxazole), 1617 (CONH str., amide), 1570 (C=N str., Pyridine); ^1^H-NMR: 7.410–8.83 (m, 12H, ArH), 4.59 (s, 2H, –CH_2_S), 4.63 (s, 2H, –NCH_2_), 7.49 (s, 1H, N=CH–Ar), 7.413 (s, 1H, –NH); ^13^C-NMR: 170.8, 165.4, 150.1, 144.3, 141.7, 139.8, 132.8, 124.8, 124.4, 119.2, 113.7, 110.9, 33.3, 29.5; MS ES + (ToF): *m/z* 443 [M^+^+1]; CHN: Calc. C_23_H_18_N_6_O_2_S: C, 62.43; H, 4.10; N, 18.99; Found: C, 62.49; H, 4.15; N, 19.05.

##### Compound 24

IR: 3057 (C–H str., aromatic), 1454 (C=C str., aromatic), 1671 (C=N, N=CH str.), 1243 (C–N str.), 709 (C–S str., CH_2_S), 1198 (C–O–C str. of oxazole), 1623 (CONH str., amide), 1570 (C–H str., thiophene); ^1^H-NMR: 7.06–8.37 (m, 8H, Ar–H), 4.62 (s, 2H, CH_2_–S), 4.59 (s, 2H, –NCH_2_), 8.26 (s, 1H, N=CH–Ar), 7.07 (s, 1H, –NH), {7.08 (d, 1H, CH), 7.54 (t, 1H, CH), 7.84 (d, 1H, CH) of thiophene ring}; ^13^C-NMR: 170, 164.8, 151.1, 141.7, 138.8, 127.7, 125.3, 124.9, 124.4, 119.2, 113.7, 110.8, 33.3, 29.6; MS ES + (ToF): *m/z* 448 [M^+^+1]; CHN: Calc. C_22_H_17_N_5_O_2_S_2_: C, 59.04; H, 3.83; N, 15.65; Found: C, 59.14; H, 3.85; N, 15.58.

##### Compound 25

IR: 2919 (C–H str., aromatic), 1454 (C=C str., aromatic), 1661 (C=N, N=CH str.), 1244 (C–N str.), 739 (C–S str., CH_2_S), 1171 (C–O–C str. of oxazole), 1626 (CONH str., amide), 1572 (C–H str., thiophene); ^1^H-NMR: 6.90–8.40 (m, 8H, Ar–H), 3.72 (s, 2H, CH_2_–S), 4.62 (s, 2H, –NCH_2_), 8.26 (s, 1H, N=CH–Ar), 7.20 (s, 1H, NH), {7.55 (d, 1H, CH), 7.77 (d, 1H, CH) of thiophene ring}, 2.52 (s, 3H, –CH_3_); ^13^C-NMR: 169.8, 164.6, 151.1, 141.7, 139.1, 137.4, 127.3, 125.3, 124.9, 124.4, 119.2, 113.7, 110.8, 33.4, 28.9, 13.4; MS ES + (ToF): *m/z* 462 [M^+^+1]; CHN: Calc. C_23_H_19_N_5_O_2_S_2_: C, 59.85; H, 4.15; N, 15.17; Found: C, 59.91; H, 4.22; N, 15.23.

##### Compound 26

IR: 3046 (C–H str., aromatic), 1457 (C=C str., aromatic), 1663 (C=N, N=CH str.), 1249 (C–N str.), 746 (C–S str., CH_2_S), 1197 (C–O–C str. of oxazole), 1618 (CONH str., amide), 3214 (–OH); ^1^H-NMR: 6.85–7.77 (m, 12H, ArH), 4.58 (s, 2H, –CH_2_S), 4.63 (s, 2H, –NCH_2_), 8.26 (s, 1H, N=CH–Ar), 7.89 (s, 1H, –NH), 4.6 (s, 1H, –OH); ^13^C-NMR: 170.1, 164.8, 151.1, 141.7, 139.8, 132.8, 131.1, 124.9, 124.4, 119.2, 118.5, 113.7, 110.9, 33.1, 29.5; MS ES + (ToF): *m/z* 458 [M^+^+1]; CHN: Calc. C_24_H_19_N_5_O_3_S: C, 63.01; H, 4.19; N, 15.31; Found: C, 63.08; H, 4.11; N, 15.38.

#### Biological study

##### Antimicrobial activity

Tube dilution method [21] was used for the determination of minimum inhibitory concentration (MIC) of the synthesized derivatives (**1**–**26**) using ofloxacin and fluconazole as standard drugs against seven microbial species i.e. *B. subtilis* (MTCC-441), *E. coli* (MTCC-443)*, P. aeruginosa* (MTCC-424)*, S. typhi* (MTCC-98)*, K. pneumoniae* (MTCC-530),

*Candida albicans* (MTCC-227) and *A. niger* (MTCC-281). Double strength nutrient broth I.P. (bacteria) or sabouraud dextrose broth I.P. (fungi) was used for the preparation of the serial dilution of test and standard compounds [23]. Dimethyl sulfoxide (DMSO) was used for the preparation of stock solution of test and standard compounds. The concentrations of 50, 25, 12.5, 6.25, 3.125 and 1.562 µg/ml were obtained by doing further progressive dilutions. The samples were incubated at 37 ± 1 °C for 24 h (bacteria), at 25 ± 1 °C for 7 days (*A. niger*) and at 37 ± 1 °C for 48 h (*C. albicans*), respectively and the results were recorded in terms of MIC. The lowest concentration of the compounds under evaluation that showed no signs of microbial growth of in the tube was the MIC. A control was performed with the test medium supplemented with DMSO at the same dilutions as used in the study to ensure that the solvent had no effect on the bacterial growth.

##### Anticancer activity

Human colorectal carcinoma [HCT-116 (ATCC (American Type Culture Collection) CCL-247)] cancer cell line was used for the determination of anticancer activity of the prepared derivatives using 2-(3-diethyl-amino-6-diethylazaniumylidene-xanthen-9-yl)-5-sulfobenzene-sulfonate (SRB) assay. In this study, trichloroacetic acid was used for fixing the cells and then staining was done for 30 min with 0.4% (w/v) sulforhodamine B mixed with 1% acetic acid. Five washes of 1% acetic acid solution helped in discarding the unbound dye and protein-bound dye was extracted with 10 mM unbuffered tris base solution for confirmation of optical density at 570 nm in a computer-interfaced, 96-well microtiter plate reader [22].

## Conclusion

A series of new benzoxazole derivatives was prepared and its chemical structure was confirmed by ^1^H/^13^C NMR, Mass and IR studies. All the derivatives were further evaluated for antibacterial, antifungal and anticancer activity and it was observed that the compounds **1**, **10**, **13**, **16**, **19**, **20** and **24** displayed the best activity against various microbial species in comparison to reference drug ofloxacin and fluconazole. In case of anticancer activity, the compound **4** was the most active whereas compounds **6** and **26** had activity closer to the reference drug, 5-fluorouracil.
